# Establishment and Application of a Real-Time Recombinase Polymerase Amplification Assay for the Detection of Avian Leukosis Virus Subgroup J

**DOI:** 10.3389/fvets.2022.847194

**Published:** 2022-07-07

**Authors:** Guanggang Qu, Yun Li, Zhongwei Zhao, Lizhong Miao, Feng Wei, Na Tang, Qingqing Xu, Venugopal Nair, Yongxiu Yao, Zhiqiang Shen

**Affiliations:** ^1^Binzhou Animal Science and Veterinary Medicine Academy and UK-China Centre of Excellence for Research on Avian Diseases, Binzhou, China; ^2^College of Veterinary Medicine, Shandong Agricultural University, Tai'an, China; ^3^Shandong Lvdu Biotechnology Co., Ltd, Binzhou, China; ^4^The Pirbright Institute and UK-China Centre of Excellence for Research on Avian Diseases, Guildford, United Kingdom

**Keywords:** Avian Leukosis Virus Subgroup J, recombinase polymerase amplification assay, real-time RPA, rapid diagnosis, on-site

## Abstract

Avian leukosis caused by avian leukosis virus (ALV), belonging to the genus *Alpharetrovirus* of the family *Retroviridae*, is associated with benign and malignant tumors in hemopoietic cells in poultry. Although several methods have been developed for ALV detection, most of them are not suitable for rapid on-site testing due to instrument limitations, professional operators, or the low sensitivity of the method. Herein, we described the real-time recombinase polymerase amplification (RPA) assay for rapid detection of ALV subgroup J (ALV-J). The major viral structural glycoprotein gp85, highly specific for the subgroup, was used as the molecular target for the real-time RPA assay. The results were obtained at 38°C within 20 min, with the detection sensitivity of 10 copies/μl of standard plasmid pMD18-T-gp85 as the template per reaction. Real-time RPA was capable of ALV-J-specific detection without cross-reaction with other non-targeted avian pathogens. Of the 62 clinical samples tested, the ALV-positive rates of real-time RPA, PCR, and real-time PCR were 66.13% (41/62), 59.68% (37/62), and 67.74% (42/62), respectively. The diagnostic agreement between real-time RPA and real-time PCR was 98.39% (61/62), and the kappa value was 0.9636. The developed real-time ALV-J assay seems promising for rapid and sensitive detection of ALV-J in diagnostic laboratories. It is suitable for on-site detection, especially in a poor resource environment, thus facilitating the prevention and control of ALV-J.

## Highlights

- Establishment of the Real-Time Recombinase Polymerase Amplification (RPA) Method for Rapid Detection of Avian Leukosis Virus Subgroup J.
- The Specificity and Sensitivity (Detection Limit of the Assay Is 10 Copies/Reaction of Standard Plasmid) of the Optimized Real-Time RPA Assay Were Evaluated.
- Clinical Application Revealed More Sensitivity of the Real-Time RPA Assay Than the Conventional PCR and Had Good Consistency With Real-Time PCR, Suggesting the Potential for Clinical Diagnosis.

## Introduction

Avian leukosis virus (ALV) is the avian leukosis/sarcoma virus that causes various tumors in chickens (1). It is divided into endogenous and exogenous viruses. The exogenous viruses infecting chickens are classified into five major subgroups, namely, A–D, and J, based on the envelope glycoprotein (gp85) identified by virus serum neutralization tests (1, 2). The ALV subgroup J (ALV-J) is a recombinant between exogenous ALVs and the family of endogenous avian retroviruses (3). The most common subgroups identified in the field are A, B, and J. ALV-J is spread by horizontal and vertical transmission and causes myeloid leukosis and other tumors in meat-type and egg-type chickens (1, 2), resulting in significant economic losses due to increased mortality, decreased productivity, and the cost for eradication. There is no commercial vaccine to prevent avian leukosis (1); thus, eradication of ALV from the breeding flocks is the primary method to control avian leucosis (4, 5). Although ALV-J has been eradicated in most Western countries, it is a major challenge for the poultry industry in Asian countries, including China (4, 6, 7).

Avian leukosis virus (ALV) eradication is based on regular monitoring and strictly eliminating the infected birds. Presently, the commonly used methods for detecting ALV include virus isolation and identification, enzyme-linked immunosorbent assay (ELISA), real-time PCR (RT-PCR), conventional PCR, immunofluorescence assay (IFA), and quantitative competitive reverse-transcription PCR (QC-RT-PCR) (8, 9). The method of virus isolation in cell culture is time-consuming as it requires a minimum of 7 days to obtain results (9–11). Antigen capture ELISA (AC-ELISA) plays a major role in eradicating ALVs (8). However, ELISA detects group-specific antigen p27 but cannot distinguish between endogenous and exogenous ALV (8, 12). Thus, RT-PCR and immunofluorescence methods for antigen detection and identification of endogenous and exogenous ALVs have been developed (8), but these tests require complicated and expensive instruments, which are not suitable for field detection of ALVs (8, 9).

In recent years, isothermal amplification methods have attracted increasing attention because of their ease of use, short duration, and independence of specialized equipment for rapid diagnosis (13). Among the isothermal amplification methods, recombinase polymerase amplification (RPA), an isothermal amplification technology, was developed as an alternative to PCR assay to amplify nucleic acids under isothermal conditions (14, 15). The RPA assay depends on several enzymes and proteins at a constant temperature, such as recombinase, single-strand binding protein, Exonuclease III, and strand displacing DNA polymerase to facilitate gene amplification (16). The recombinase binds to the primer to form a complex and searches for the homologous sequences in the double-stranded DNA. Subsequently, the chain exchange reaction is initiated to form and initiate DNA synthesis, and the target region on the template is amplified exponentially. The replaced DNA strand binds to the single-stranded binding protein, preventing further replacement (17). One significant advantage of RPA is that the amplifications could be detected by agarose gel electrophoresis (AGE) and a real-time fluorescence detection platform (18) or visualized by a lateral flow dipstick (LFD) assay (19). Moreover, RPA has the advantage of amplification at a low temperature (37–42°C), with a 20-min reaction time, which is considerably shorter than many isothermal techniques. Different types of RPA-based assays have been successfully used in the detection of pathogens in plants (20, 21), animals (19, 22, 23), and humans (24–26). Thus, the present study aimed to establish a real-time RPA assay with an exo probe for rapid, specific, and sensitive detection of ALV-J in the field.

## Materials and Methods

### Virus Strains and Clinical Samples

Archived ALV viral DNA from different subgroups, namely, J (ALV-J), A (ALV-A), B (ALV-B), D (ALV-D), and K (ALV-K), preserved in the key laboratory of Shandong Binzhou Animal Science and Veterinary Medicine Academy, were used in this study. Various batches of commercial lyophilized live vaccines include Newcastle disease bivalent vaccines (Batch No. 007, 142, 152, and 063), Newcastle disease live vaccines (Batch No. 145 and 179), live pox vaccines (Batch No. 134 and 147), chicken infectious bursal disease vaccines (Batch No. 118 and 124), and live duck plague vaccines (Batch No. 153 and 177). IBDV, IBV, ILTV, NDV, and AIV H9 viruses were isolated from the clinical samples. The suspected ALV samples were collected from the chicken farms in Shandong Province.

### Viral Nucleic Acid Extraction

An equivalent of 50 mg of each tissue sample was homogenized in 200 μl phosphate-buffered saline (PBS), and the supernatant was collected by centrifugation at 3,000 g, 4°C for 10 min for DNA extraction. Vaccine samples were resuspended in 1 ml of PBS for DNA/RNA extraction. DNA/RNA was extracted using Axygen^®^ AxyPrep™ Body Fluid Viral DNA/RNA Miniprep Kit (Axygen BioScience, Inc., USA), according to the manufacturer's instructions. The RNA extracted from the RNA virus vaccines was reverse transcribed into cDNA (Life Technologies, USA). All templates were stored at −80°C until further analysis.

### RPA Primers and Exo Probe Designing

The genome sequences of ALV-J (GenBank number: Z46390.1, KP284572.1, KC149972.1, KC149971.1, FJ216405.1, AF307949.1, and AF307950.1), ALV-A (GenBank number: M37980.1), ALV-B (GenBank number: AF052428.1), ALV-C (GenBank number: J02342.1), ALV-D (GenBank number: D10652.1), and ALV-E strains (GenBank number: DQ412728.1 and DQ412729.1) were retrieved from GenBank and aligned using DNAStar software (DNASTAR, Madison, USA). The sequence of ALV-J gp85 had a low homology to that of other exogenous subgroups (3), making it an ideal molecular target for the real-time RPA. The design of the primers and the probe of RPA is crucial for the specificity and amplification efficacy. Since there is no optimal design software available, all the primers and the probe of RPA were designed using Primer Premier 5.0, following the instructions of TwistDX. To avoid the formation of the primer's hairpin structure and dimers, the RPA primer design should avoid multiple guanines at the 5' end and ensure that the GC content is 30–70%. Furthermore, to ensure the sensitivity of the detection and the rapid progress of the reaction, the length of the RPA product was set to 100–200 bp.

The forward and reverse primers and the exo probe were designed according to the gp85 sequences following the RPA manufacturer's guidelines (TwistDx. Cambridge, UK). The primers are listed in Table 1. The primers and the probe were analyzed and screened using NCBI Primer-Blast to ensure specificity. All primers and the probe were synthesized by Sangon (Shanghai, China).

**Table 1:** Primers and probe of real-time RPA.

**Name**	**Sequence (5^**′**^−3^**′**^)**	**Location**
ALV-J-F1	CAATCATGGACGATGGTAAGTCCAATAAAC	726–755
ALV-J-F2	RTTGCGTGACTTCATAGVAAAATGGAAAAG	666–695
ALV-J-F3	CTATGTCAACCAATCATGGACGATGGTAAG	717–746
ALV-J-R1	AGCCCTGTCCCCACAAATCAAGAAAATACC	1,021–1,050
ALV-J-R2	ATTYTGTCCCRTTRCTGTAYCCCGCTGACC	863–892
ALV-J-R3	CAAGCCCTGTCCCCACAAATCAAGAAAATA	1,023–1,052
ALV-J-R4	CGAAGGTAAACCCATATGCATAATAATTCCATTC	934–963
ALV-J-R5	CCTCCCAAGGCATTACGCGGGATGCCTTGC	1,053–1,082
ALV-J-probe	TAGATATTGTGGATTCACCAGYAACGAGAC[FAM-dT] [THF]G[BJQ1-dT]TAYTATMGAGGGRAC-C3 Spacer	780–828

### Construction of Standard DNA Plasmid

*ALV-J-gp85* gene was amplified by PCR using forward primer 5'-GACTTCATAGVAAAATGGAAAAG-3' and reverse primer 5'-CTGTCCCCACAAATCAAGAAAATA-3'. The standard DNA plasmid pMD18-T-gp85 was obtained by cloning purified gp85 PCR product into plasmid pMD18-T according to the manufacturer's instructions for pMD18-T Vector Cloning Kit (Takara Bio Inc., Japan). The concentration of the constructed plasmids was measured on a NanoDrop 2000c spectrophotometer (Thermo Scientific, USA) at 260 nm. The copy number of the plasmid was calculated using the following formula: Number of copies = (amount of target DNA in nanograms) × Avogadro's number (6.0221 × 10^23^) / length of DNA amplicon in base pair (bp) × 660 × (1 × 10^9^) (27). The generated DNA standard was used as an initial template in the sensitivity analysis.

### Real-Time RPA Assay

The exo-RPA reaction was performed in 50 μl using a TwistAmp™ exo kit (TwistDX, UK). A master mixture was prepared according to the manufacturer's instructions. The mixture contained 25 μl 2 × reaction buffer, 8.2 μl dNTPs (1.8 μM), 5 μl 10 × Probe E-mix, 2.1 μl forward Primer (10 μM), 2.1 μl reverse primer (10 μM), 0.6 μl TwistAmp™ exo probe (10 μM), 2.5 μl 20 × Core Reaction Mix, 1 μl 50 × EXO. Subsequently, 2.5 μl of 280 mM magnesium acetate and 1 μl nucleic acid were added to the mixture to initiate the amplification reaction on the LightCycler 480 Instrument II (Roche Diagnostics Corporation, IN, USA) for 40 cycles at 38°C for 20 min (30 s/cycle).

### Sensitivity and Specificity Analysis

To determine the sensitivity of the RPA assay, 10-fold serial dilutions of pMD18-T-gp85 standard plasmid ranging from 10^6^ to 10^0^ copies/μl were tested. All the samples and positive/negative controls were evaluated in duplicate. The specificity of the assay was assessed using other viral pathogens of chicken. A panel of viruses, including ALV-J, ALV-A, ALV-B, ALV-D, ALV-K, IBDV, ILTV, IBV, NDV, and AIV H9, were significant pathogens of poultry and hence utilized in the test. Also, the positive and negative controls were tested simultaneously. The RNA extracted from RNA viruses, such as IBDV, IBV, NDV, and AIV H9, was reverse transcribed into cDNA (Life Technologies) for subsequent RPA test.

### Conventional PCR

ALV-J conventional PCR was developed for the detection of ALV-J in a 25 μl reaction using primers PF: 5'-CGGAGAAGACACCCTTGCT-3'and JR: 5'-CGAACCAAAGGTAACACACG-3', as described previously [Gao et al., (8)]. Briefly, the reaction mixture consisted of 12.5 μl of 2 × Premix Taq (TaKaRa, China), 2 μl forward primer PF (10 μM) and 1 μl reverse primer JR (10 μM), 2 μl of sample DNA, and the appropriate volume of DNase-free water.

The PCR reaction was as follows: initial denaturation at 94°C for 5 min, followed by 30 cycles at 94°C for 30 s for denaturation, 56°C for 30 s for annealing, and 72°C for 1 min for extension, and a final extension at 72°C for 1 min. To confirm the results, all specific fragments amplified from the clinical samples by RPA and conventional PCR were purified using a DNA gel extraction kit (Omega Bio-Tek, Inc., GA, USA) and sequenced by Sangon.

### Reproducibility of the RPA Assay

The reproducibility of the assay was confirmed using three dilutions of standard plasmid pMD18-T-gp85. To determine the interassay variations, each sample was tested in triplicate using 2 μl of each plasmid per reaction. The assay was also repeated three times to evaluate the intraassay variations. The coefficients of variation (CVs) for Ct values of the intra- and interassay comparisons were determined.

### Real-Time RPA Assay Validation

To validate the developed assay, 50 clinical samples collected from several poultry flocks and 12 different batches of poultry lyophilized vaccines, randomly purchased from the licensed companies in China, were examined. RPA was also performed concurrently with conventional PCR and real-time PCR, as described previously (8, 9). The total agreement of RPA assay with conventional PCR and real-time PCR was verified. The calculation of the kappa value has been reported previously (28).

## Results

### Real-Time RPA Primer Screening

**Figure 1:**
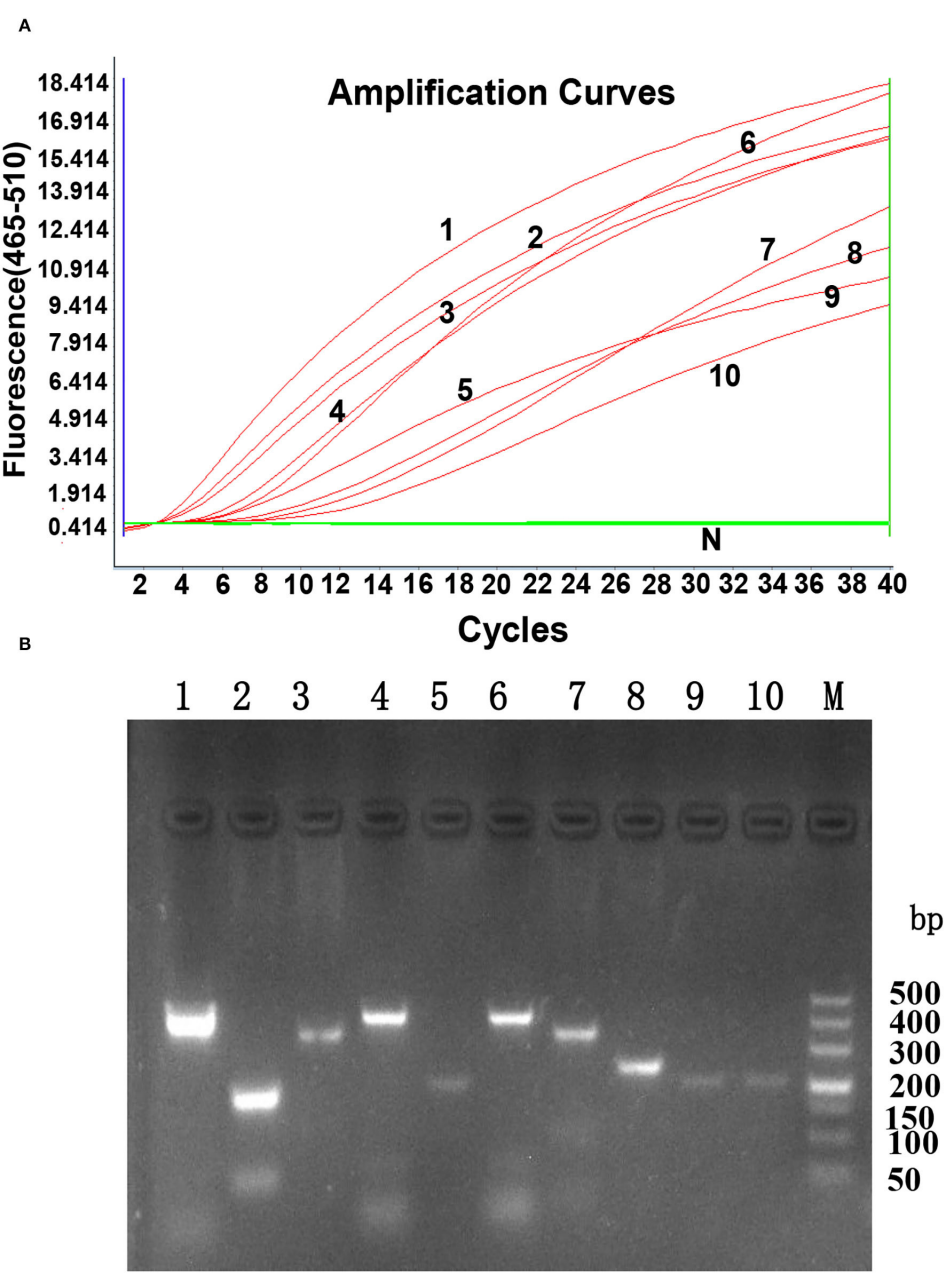
The generated amplification plot for different combinations of primers and probes in real-time RPA. **(A)**. Amplification curve by LightCycler 480 Instrument II. N: Negative control; 1: ALV-J F1-R1; 2: ALV-J F1-R2; 3: ALV-J F1-R5; 4: ALV-J F2-R1; 5: ALV-J F2-R2: 6: ALV-J F2-R3; 7: ALV-J F3-R3; 8: ALV-J F3-R4; 9: ALV-J F3-R5; 10: ALV-J F3-R1. Reactions were scored positive when the change in fluorescence exceeded 1.0 units. **(B)**. Nucleic acid detection on agarose gel electrophoresis. 1: ALV-J F1-R1; 2: ALV-J F1-R2; 3: ALV-J F1-R5; 4: ALV-J F2-R1; 5: ALV-J F2-R2: 6: ALV-J F2-R3; 7: ALV-J F3-R3; 8: ALV-J F3-R4; 9: ALV-J F3-R5; 10: ALV-J F3-R1; M: DL500 DNA marker.

According to the principles of RPA primer design, three forward primers, five reverse primers, and one RPA exo probe were designed, as shown in Table 1. The oligonucleotide backbone of the probe includes an inverse arrangement of fluorophore [6-carboxyfluorescein (FAM)], quencher [black hole quencher 1 (BHQ-1)], internal abasic site mimic [tetrahydrofuran spacer (THF)], and a 3'-polymerase extension blocking group C3-spacer. The primers and probe were assessed using the TwistAmp exo kit according to the manufacturer's instructions. All ten primer sets successfully generated the amplification curve within 20 min, as shown in Figure 1. The best primer pair ALV-J-F1/R1 with the highest amplification efficiency was identified and used for further analysis. The data are shown in Table 2.

**Table 2:** Screening of the best real-time RPA primer pairs.

**No**.	**Primer pairs**	**Ct**	**Fluorescence signal reading (wavelength: 465–510 nm)**
1	ALV-J F1-R1	5.12	18.032
2	ALV-J F1-R2	5.78	16.884
3	ALV-J F1-R5	5.85	16.509
4	ALV-J F2-R1	7.56	18.759
5	ALV-J F2-R2	8.24	11.259
6	ALV-J F2-R3	6.92	16.884
7	ALV-J F3-R3	11.05	14.259
8	ALV-J F3-R4	9.89	12.759
9	ALV-J F3-R5	12.54	10.134
10	ALV-J F3-R1	6.35	8.259

### Sensitivity of the Real-Time RPA Assay

**Figure 2:**
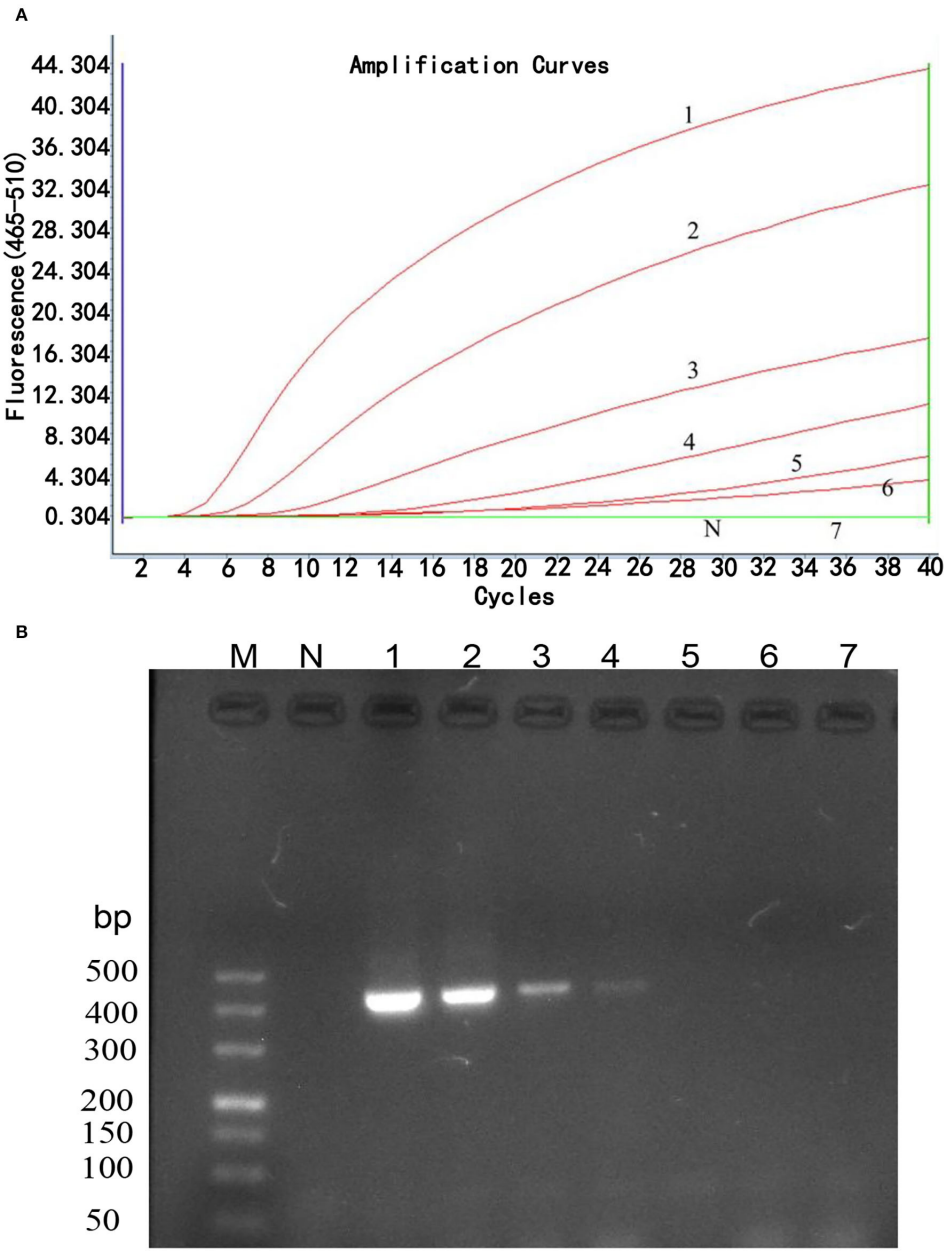
Sensitivity analysis of real-time RPA assay. Serial dilutions of plasmid pMD18-gp85 DNA (10^6^-10^0^ copies corresponding to curves 1–7) were used as the templates for real-time RPA reactions. **(A)**. Sensitivity analysis by LightCycler 480 Instrument II. The detection limit was 10 copies of DNA/reaction for the real-time RPA assay (curve 6). Reactions were scored positive when the change in fluorescence exceeded 1.0 units. Curve N used nuclease-free water as a negative control. **(B)**. Sensitivity analysis by electrophoresis. M: DL500 DNA marker; 1: 1× 10^6^ copies/μl; 2: 1× 10^5^ copies/μl; 3: 1× 10^4^ copies/μl; 4: 1× 10^3^ copies/μl; 5: 1× 10^2^ copies/μl; 6: 1× 10^1^ copies/μl; 7: 1× 10^0^ copies/μl.

The sensitivity of the real-time RPA assay was assessed by testing 10-fold serial dilutions of DNA standards ranging from 1 × 10^6^ to 1 × 10^0^ copies/μl. The results showed (Figure 2A) that the detection limit of the real-time RPA assay was 10 copies/μl of *gp85* gene per reaction by exo probe on a LightCycler 480 Instrument II. In contrast, the detection limit of the real-time RPA assay was 10^3^ copies/μl of *gp85* gene per reaction, as analyzed by AGE and ethidium bromide staining (Figure 2B). The results demonstrated that the sensitivity of the established real-time RPA assay with an exo probe was 100 times higher than that of the real-time RPA assay.

### Specificity of Real-Time RPA

**Figure 3:**
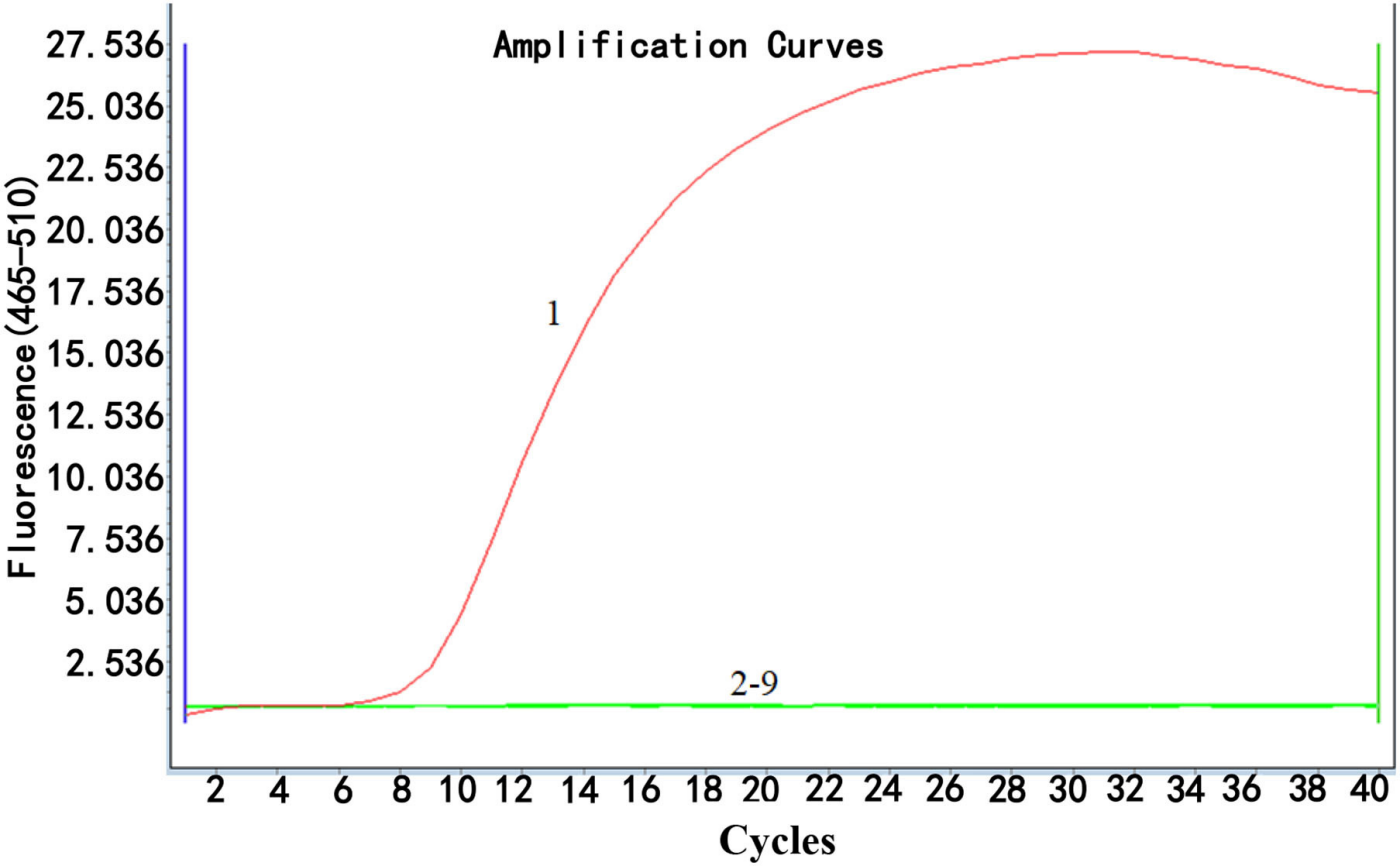
Specificity analysis of real-time RPA assay. Only DNA from ALV-J infected samples was positively amplified (curve 1), while no amplification of other DNA or RNA viruses (cDNA) (curves 2–9 were from the following viruses: ALV-A, ALV-B, ALV-D, ALV-K, IBDV, ILTV, IBV, NDV, and H9, respectively) was observed. Nuclease-free water was used for curve N as a negative control. The results suggested that the developed real-time RPA assay was highly specific. Reactions were scored positive when the change in fluorescence exceeded 1.0 units.

The specificity of real-time RPA was determined by examining the ability of the method to detect ALV-J and differentiate it from ALV-A, ALV-B, ALV-D, ALV-K, and other common avian viruses, namely, IBDV, ILTV, IBV, NDV, and H9. As shown in Figure 3, the fluorescence signal was only detected from ALV-J, while no fluorescence signal was observed from other viruses, demonstrating high specificity of the real-time RPA assay for ALV-J detection without cross-reactions with other subgroups of ALVs and other common avian viruses.

### Reproducibility of Real-Time RPA

**Figure 4:**
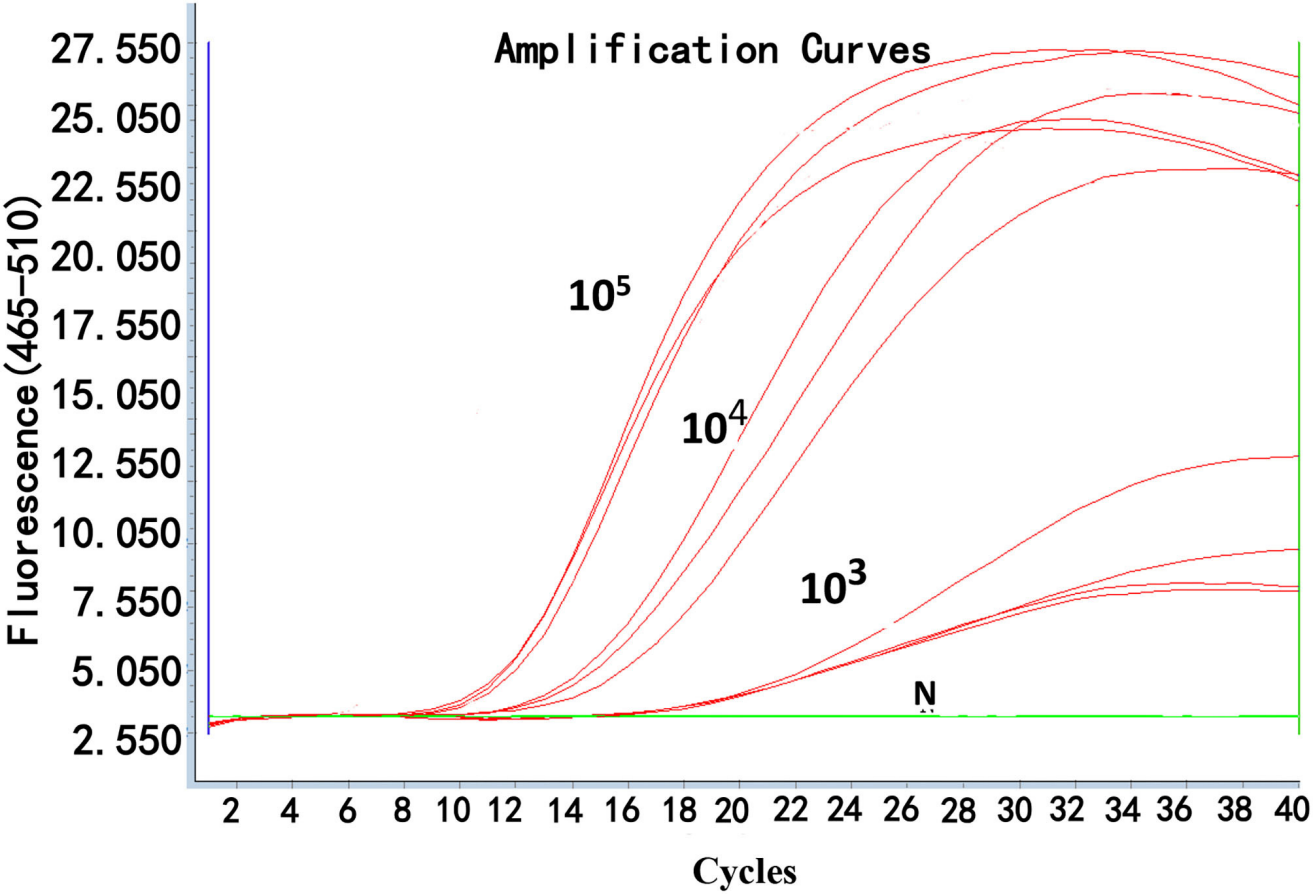
Reproducibility of real-time RPA.

To determine the reproducibility of real-time RPA, the interassay and intraassay of reproducibility were assessed using testing standard plasmids (10^5^, 10^4^, and 10^3^ copies/μl) three times independently (Figure 4). Also, the SDs were calculated. The inter-CV and intra-CV values ranged from 0.05 to 0.16% and 0.49 to 2.69%, respectively, indicating that the assay was highly reproducible (Table 3).

**Table 3:** Intrareproducibility and interreproducibility assay.

**Dilution of plasmid**	**Interassay**					**Intraassay**				
	**Ct value**				**CV%**	**Ct value**				**CV%**
	**Assay 1**	**Assay 2**	**Assay 3**	**Average SD**		**Assay 1**	**Assay 2**	**Assay 3**	**Average SD**	
10^5^	9.87	9.60	9.89	0.16	1.65%	9.64	9.15	9.51	0.25	2.69%
10^4^	11.59	12.18	11.72	0.31	2.62%	11.49	11.56	11.42	0.07	0.61%
10^3^	17.45	17.51	17.55	0.05	0.29%	17.61	17.78	17.67	0.09	0.49%

### Comparison of Real-Time RPA Assay, Conventional PCR Assay, and Real-Time PCR Assay in Clinical Samples and Commercial Vaccine Detection

**Figure 5:**
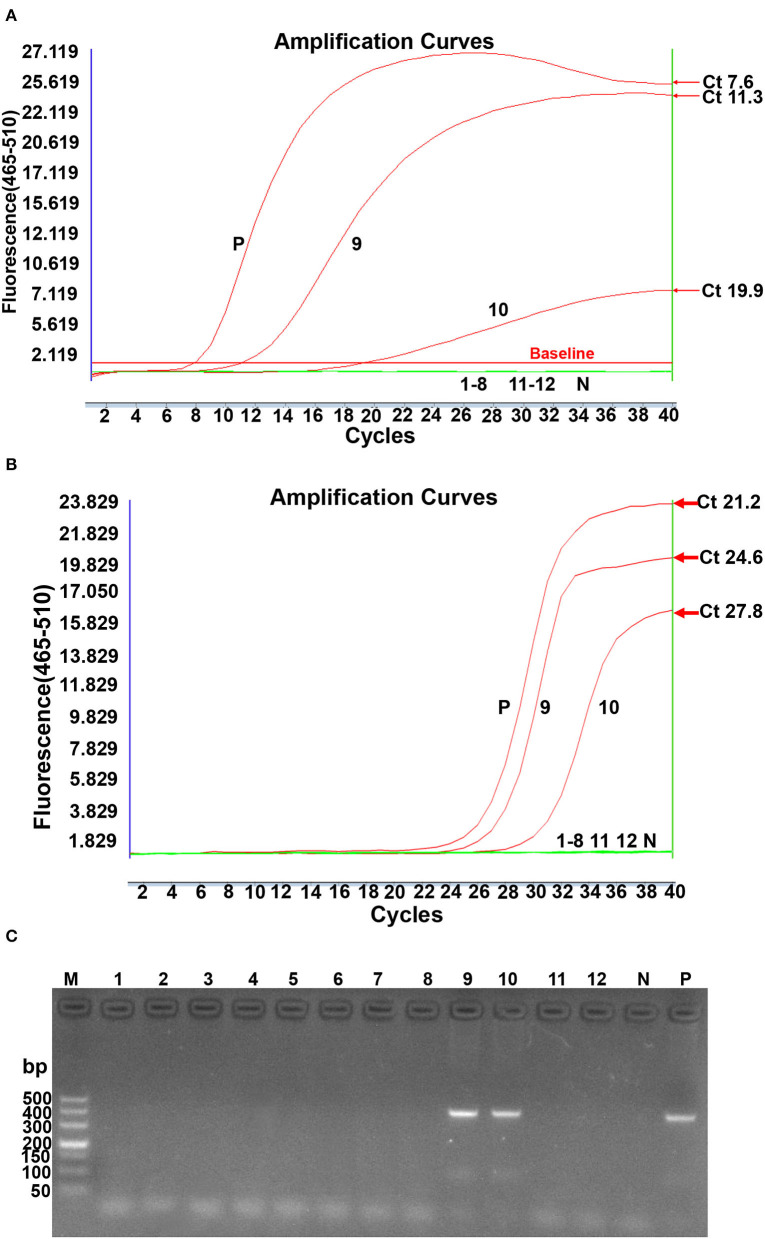
ALV subgroup J contamination of lyophilized vaccines detected by real-time RPA, real-time PCR, and conventional PCR simultaneously. **(A)**. Real-time RPA detection. N: Negative; P: Positive; 1–8 lanes: Newcastle disease bivalent vaccines (Batch No. 007, 142, 152, and 063), Newcastle disease live vaccines (Batch No. 145 and 179), live duck plague vaccines (Batch No. 153 and 177); 9: fowl pox live vaccine 134; 10: fowl pox live vaccine 147; 11–12: infectious chicken bursa (Batch No. 118 and 124). **(B)**. Real-time PCR detection. N: Negative; P: Positive: 1~8 lanes: Newcastle disease bivalent vaccines (Batch No: 007, 142, 152, and 063), Newcastle disease live vaccines (Batch No: 145 and 179), Live duck plague vaccines (Batch No: 153 and 177); 9: Fowlpox live vaccine 134; 10: Fowlpox live vaccine 147; 11-12: chicken infectious bursa (Batch No: 118 and 124). Reactions were scored positive when the change in fluorescence exceeded 1.0 units. **(C)**. Conventional PCR detection. M: DL500 DNA marker; 1~8 lanes: Newcastle disease bivalent vaccines (Batch No: 007, 142, 152, and 063), Newcastle disease live vaccines (Batch No: 145 and 179), Live duck plague vaccines (Batch No: 153 and 177); 9: Fowlpox live vaccine 134; 10: Fowlpox live vaccine 147; 11–12: chicken infectious bursa (Batch No: 118 and 124); N: Negative; P: Positive.

The clinical performance of the real-time RPA assay was evaluated by testing 50 suspected clinical samples and 12 batches of commercial vaccines and compared to conventional PCR and real-time PCR methods. As shown in Table 4, 39 of these clinical samples were ALV-positive by real-time RPA method, 35 were ALV-positive by conventional PCR method, and 40 samples were ALV-positive by real-time PCR method. The commercial vaccine test results showed that 2 samples were ALV-positive and 10 vaccine samples were negative, as assessed by the real-time RPA method (Figure 5A), which was consistent with the findings of conventional PCR (Figure 5C) and real-time PCR (Figure 5B). The ALV-positive rates of real-time RPA, PCR, and real-time PCR were 66.13% (41/62), 59.68% (37/62), and 67.74% (42/62), respectively. Among the 62 samples, only 1 tested negative for ALV by real-time RPA method but was positive by real-time PCR method. The diagnostic agreement between real-time RPA and real-time PCR was 98.39% (61/62) (Table 4), and the kappa value was 0.9636. Therefore, based on these test results, it could be inferred that the real-time RPA assay of ALV-J is more sensitive than the conventional PCR assay and has good consistency with real-time PCR, indicating its potential for clinical diagnosis.

**Table 4:** Agreement between real-time RPA detection, real-time PCR, and conventional PCR.

**Sr. No**	**Sample**	**Real-time RPA**	**Real-time PCR**	**Conventional PCR**
1	Heart	+	+	+
2	Heart	+	+	+
3	Heart	–	+	–
4	Heart	–	–	–
5	Heart	+	+	–
6	Heart	+	+	+
7	Heart	+	+	+
8	Heart	+	+	+
9	Heart	+	+	+
10	Heart	+	+	+
11	Heart	+	+	+
12	Heart	+	+	+
13	Heart	+	+	+
14	Spleen	–	–	–
15	Spleen	–	–	–
16	Spleen	–	–	–
17	Spleen	+	+	+
18	Spleen	+	+	–
19	Spleen	+	+	+
20	Spleen	+	+	+
21	Spleen	+	+	+
22	Liver	–	–	–
23	Liver	–	–	–
24	Liver	–	–	–
25	Liver	+	+	+
26	Liver	–	–	–
27	Liver	+	+	+
28	Liver	+	+	+
29	Liver	–	–	–
30	Liver	+	+	–
31	Liver	+	+	+
32	Liver	+	+	+
33	Liver	+	+	+
34	Liver	+	+	+
35	Bursa	+	+	+
36	Bursa	+	+	+
37	Bursa	+	+	+
38	Bursa	+	+	–
39	Bursa	+	+	+
40	Bursa	+	+	+
41	Bursa	+	+	+
42	Bursa	+	+	+
43	Kidney	+	+	+
44	Kidney	+	+	+
45	Kidney	+	+	+
46	Kidney	+	+	+
47	Kidney	+	+	+
48	Kidney	+	+	+
49	Kidney	+	+	+
50	Kidney	–	–	–
51	NDV Vaccine-007	–	–	–
52	NDV Vaccine-142	+	+	+
53	NDV Vaccine-152	–	–	–
54	NDV Vaccine-063	–	–	–
55	NDV Vaccine-145	–	–	–
56	NDV Vaccine−179	–	–	–
57	Duck plague-153	–	–	–
58	Duck plague-177	+	+	+
59	Fowlpox live vaccine-134	–	–	–
60	Fowlpox live vaccine-147	–	–	–
61	IBD vaccine-118	–	–	–
62	IBD vaccine-124	–	–	–

## Discussion

Since 2008, ALV-J has caused severe economic losses owing to the induction of various malignant tumors and other reproductive problems in birds in China (8). Due to the high level of vertical and horizontal transmission of ALV-J and the lack of effective vaccines and drugs to prevent this disease (4, 29, 30), it is essential to improve the efficiency of eradication and accelerate the elimination process.

Real-time RPA assay has been widely utilized to detect human and animal pathogens (31, 32). The current study describes specific, sensitive, and rapid ALV-J methods based on exo probe real-time RPA assay, which provides accurate results in 20 min at the isothermal conditions without elaborate methods for the detection of the amplified productions (33). Although RPA has the advantage of rapid reaction, it still needs the laboratory operation step of nucleic acid extraction when used in the field. The nucleic acid quick extraction reagents currently available in the market are easy to operate, and the extraction can be completed within 5 min of heating. To better apply this method to rapid on-site detection, we will combine the RPA method with the nucleic acid direct extraction method in the next step. The entire detection process would require only 25 min, providing rapid on-site detection.

Since the ALV-J *gp85* gene shows approximately 40% identity with the corresponding regions of other exogenous ALV subgroups (3). The ALV-J real-time RPA primers and probes were designed based on the sequence alignment of *gp85* genes with different strains of the ALV-J subgroup. The specificity results showed that the ALV-J real-time RPA did not amplify other exogenous subgroups (ALV-A, ALV-B, ALV-B, and ALV-K) and other common avian viruses. Therefore, this ALV-J real-time RPA using ALV-J *gp85* as the target gene showed good subgroup specificity. We also established a highly sensitive subgroup-specific real-time RPA assay for ALV-J, which can detect 10 copies/reaction. The validation of clinical samples and commercial vaccines demonstrated that the real-time RPA assay was practical in the laboratory and more sensitive than the conventional PCR assay. Additional studies are required to test more clinical samples from natural outbreaks and confirm that the RPA assay has potential for epidemiological surveillance and facilitates the eradication of ALV-J in the poultry industry and vaccine companies. Although in the current study, a Roche LightCycler 480 Instrument was used to develop RPA, the assay can also realize on-site diagnosis of clinical samples using a point-of-care (POC) instrument, Thermostatic Fluorescent Biosensor (Xianda, China) with fluorescence detection in the FAM channel (excitation 470 nm and detection 520 nm). This equipment is designed for cost-efficiency, accessibility, and the potential for scale-up. Although the sensitivity of the real-time RPA is still lower than the limit of real-time PCR, the advantages of this assay are obvious.

In summary, a rapid, reliable, simple real-time RPA assay has been developed for the detection of ALV-J. This assay is promising for specific and sensitive detection of ALV-J in diagnostic laboratories and suitable for on-site detection, deeming it a robust diagnostic tool to aid in the detection of ALV-J and subsequently decrease the rate of ALV-J transmission, thus reducing the economic impact on the poultry drastically.

## Data Availability Statement

The datasets presented in this study can be found in online repositories. The names of the repository/repositories and accession number(s) can be found in the article/supplementary material.

## Ethics Statement

The animal study was reviewed and approved by the Animal Care and Use Committee of Binzhou Animal Science and Veterinary Medicine Academy.

## Author Contributions

GQ and YL designed the primers and probes and optimized the reaction conditions and wrote the manuscript. GQ, YY, and ZS designed the experiments and revised the manuscript. LM, FW, and NT collected the clinic samples. ZZ and QX tested the diagnostic method. VN reviewed the manuscript and polished the English. All authors contributed to the article and approved the submitted version.

## Funding

This work was supported by the National Natural Science Foundation of China (grant No. 31761133002), the Biotechnology and Biological Sciences Research Council (BBSRC) Newton Fund grant BB/R012865/1, BBSRC Newton Fund Joint Centre Awards on UK-China Centre of Excellence for Research on Avian Diseases and 2017 China-UK Double-Hundred Talent Plan of Shandong Province.

## Conflict of Interest

ZZ and ZS were employed by Shandong Lvdu Biotechnology Co., Ltd. The remaining authors declare that the research was conducted in the absence of any commercial or financial relationships that could be construed as a potential conflict of interest.

## Publisher's Note

All claims expressed in this article are solely those of the authors and do not necessarily represent those of their affiliated organizations, or those of the publisher, the editors and the reviewers. Any product that may be evaluated in this article, or claim that may be made by its manufacturer, is not guaranteed or endorsed by the publisher.
